# GV1001 Reprograms CD47 Immune Checkpoint to Restore Macrophage Antitumor Activity in Oral Squamous Cell Carcinoma

**DOI:** 10.3390/ijms27073340

**Published:** 2026-04-07

**Authors:** Wei Chen, Seojin Kim, Cheyenne Beheshtian, Angela Jun, Sangjae Kim, No-Hee Park

**Affiliations:** 1 The Shapiro Family Laboratory of Viral Oncology and Aging Research, UCLA School of Dentistry, 714 Tiverton Ave, Los Angeles, CA 90095, USA; chenwei304@ucla.edu (W.C.); sj25kim@gmail.com (S.K.); cbeheshtian@dentistry.ucla.edu (C.B.); angelajun824@gmail.com (A.J.); 2 Teloid Inc., 920 Westholme Avenue, Los Angeles, CA 90024, USA; sjkim@teloid.com; 3 UCLA Jonsson Comprehensive Cancer Center, 10833 Le Conte Ave, Los Angeles, CA 90095, USA; 4 Department of Medicine, David Geffen School of Medicine at UCLA, 10833 Le Conte Ave, Los Angeles, CA 90095, USA

**Keywords:** GV1001, CD47, phagocytosis, cisplatin, oral squamous cell carcinoma

## Abstract

Cluster of Differentiation 47 (CD47) functions as a key “don’t-eat-me” signal that enables cancer cells to evade macrophage-mediated immune clearance. GV1001, a 16-amino-acid peptide derived from human telomerase reverse transcriptase (hTERT), has been reported to exhibit antitumor and anti-inflammatory properties and to downregulate CD47 expression in human cells. In this study, we investigated whether GV1001 modulated CD47 expression and enhanced antitumor immunity in oral squamous cell carcinoma (OSCC). In vitro, GV1001 significantly reduced CD47 expression in both murine and human OSCC cells in dose- and time-dependent manners, resulting in a marked increase in macrophage-mediated phagocytosis. Mechanistically, GV1001 suppressed CD47 promoter activity and inhibited multiple upstream regulator expression in murine and human OSCC cell lines, while exerting minimal effects on normal human keratinocytes and fibroblasts. In vivo, GV1001 significantly inhibited tumor growth, suppressed CD47 expression, increased macrophage infiltration, and induced tumor cell necrosis and apoptosis in both murine OSCC syngeneic graft model and human OSCC xenograft model. GV1001 administered alone or in combination with cisplatin produced antitumor effects. Collectively, these findings demonstrate that GV1001 functions as a potent immunomodulatory anticancer peptide that downregulates CD47 expression and restores macrophage-mediated tumor clearance, highlighting its potential as a therapeutic strategy for OSCC.

## 1. Introduction

Oral squamous cell carcinoma (OSCC) is the sixth leading cause of cancer-related mortality worldwide and is characterized by early metastasis, frequent recurrence, and persistently low survival rates [1]. Despite advances in diagnosis and therapy, the five-year survival rate remains poor [2]. Cisplatin, a first-line chemotherapeutic agent for advanced OSCC, induces DNA damage through the formation of cisplatin-DNA adducts that trigger cell-cycle arrest and apoptosis. However, the clinical efficacy of cisplatin is limited to a subset of patients, as many tumors acquire or exhibit intrinsic chemoresistance, ultimately leading to treatment failure [3]. Consequently, strategies that enhance cisplatin sensitivity are urgently needed.

OSCC cells evade immune destruction not only by escaping T-cell-mediated surveillance but also by suppressing innate immune mechanisms such as macrophage-mediated phagocytosis [4]. A central regulator of this process is Cluster of Differentiation 47 (CD47), a cell-surface “don’t-eat-me” signal that binds signal regulatory protein α (SIRPα) on macrophages, thereby blocking phagocytic clearance [5,6]. CD47 is frequently upregulated in OSCC and associates with poor clinical outcomes, positioning it as a compelling therapeutic target [7,8]. However, cisplatin treatment has been reported to increase CD47 expression in cancer cells, potentially contributing to its diminished therapeutic efficacy [9]. Thus, approaches capable of suppressing CD47 or its upstream regulators may augment the therapeutic efficacy of cisplatin.

GV1001 is a 16-amino acid peptide derived from human telomerase reverse transcriptase (hTERT) residues 611–626 [10,11,12,13,14]. Initially developed as a telomerase-targeted cancer vaccine, GV1001 activates antitumor immune responses against telomerase-expressing cells, reflecting the enzyme’s high activity in cancer and minimal expression in most normal tissues [15,16]. Beyond its immunologic functions, GV1001 inhibits heat shock protein 70/90 (HSP70/90) [17], hypoxia-inducible factor-1alpha (HIF-1α), and vascular endothelial growth factor (VEGF), thereby impairing angiogenesis, survival under hypoxic stress, and tumor invasiveness [18,19,20]. GV1001 has been clinically evaluated in several malignancies, including pancreatic, non-small cell lung, and colorectal cancers, often in combination with chemotherapy or immune adjuvants [21,22,23]. We previously observed that GV1001 suppressed CD47 expression in arterial smooth muscle cells and inhibited foam cell formation, which shared some pathological features with benign vascular neoplasms [12]. These observations raise the possibility that GV1001 may down-regulate CD47 in OSCC cells and thereby augment macrophage-mediated tumor clearance. Thus, we investigated whether GV1001 exerted intrinsic antitumor activity against OSCC through regulating CD47 expression.

## 2. Results

### 2.1. Effect of GV1001 on CD47 Expression and Macrophage-Mediated Phagocytosis of Mouse Oral Cancer 1 (MOC1)

**Figure 1:**
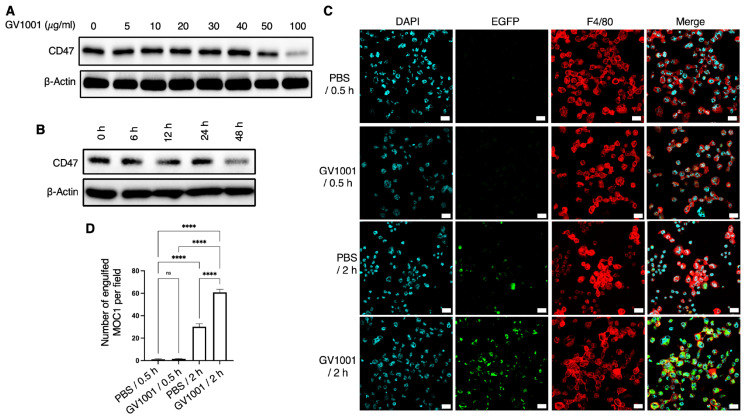
GV1001 mitigated CD47 expression and induced macrophage-mediated phagocytosis of MOC1. (**A**) Western blot analysis of CD47 levels in MOC1 cells exposed to GV1001 with varying concentrations for 48 h. β-actin level was used as a loading control. (**B**) Time-dependent effect of GV1001 (100 μg/mL) on CD47 level in MOC1 cells. (**C**) Representative immunofluorescence images of co-cultures of GV1001-pretreated MOC1 cells and macrophages following 0.5 or 2 h of incubation. Cell nuclei were stained with 4′,6-diamidino-2-phenylindole (DAPI) (blue). Enhanced green fluorescent protein (EGFP) fluorescence (green) indicates MOC1 cancer cells, whereas macrophages were stained red with an anti-F4/80 antibody. (**D**) Quantification of macrophage-mediated phagocytosis of MOC1 cells per field, analyzed using ImageJ software (v1.54). Data are presented as the mean ± standard error of the mean (SEM). Statistical analyses were performed using one-way Analysis of Variance (ANOVA) followed by Tukey’s post hoc test (ns, not significant, **** *p* < 0.0001). All experiments were performed with *n* = 5 independent biological replicates.

To determine whether GV1001 modulates CD47 expression and thereby affects macrophage-mediated phagocytosis of MOC1 cells in vitro, cells were treated with increasing concentrations of GV1001 for 48 h. As shown in Figure 1A, GV1001 decreased CD47 expression in a dose-dependent manner, with significant suppression observed at 100 μg/mL. Consistent with this finding, time-course analysis revealed that treatment with GV1001 (100 μg/mL) resulted in a progressive reduction of CD47 expression over time (Figure 1B). These data indicate that GV1001 markedly suppressed CD47 expression in MOC1 cells in a dose- and time-dependent manner. As shown in Figure 1C,D, GV1001 pretreatment significantly enhanced macrophage-mediated phagocytosis of MOC1 after 2 h of co-culture. In contrast, minimal phagocytic activity was observed at the 30 min time point. These results demonstrate that GV1001 enhances macrophage-mediated phagocytosis of MOC1 cells, at least in part through the downregulation of CD47 expression.

### 2.2. Effect of GV1001 on the CD47 Expression and Macrophage-Mediated Phagocytosis of Human OSCC Cells

**Figure 2:**
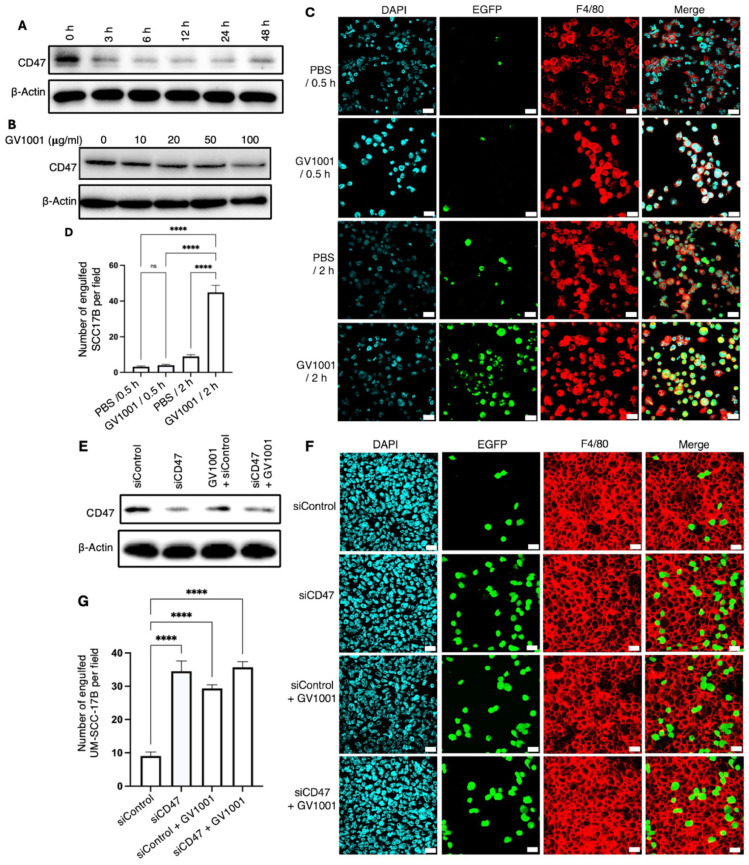
GV1001 suppressed CD47 expression and enhanced macrophage-mediated phagocytosis of UM-SCC-17B cells. (**A**,**B**) Western blot analysis of CD47 expression in UM-SCC-17B cells following treatment with GV1001 at the indicated time points and concentrations. β-Actin was used as a loading control. (**C**) Representative immunofluorescence images of co-cultures of GV1001-pretreated UM-SCC-17B cells and macrophages after 0.5 or 2 h of incubation. (**D**) Quantification of EGFP-labeled UM-SCC-17B cells engulfed by macrophages per field, analyzed using ImageJ software. (**E**) Western blot analysis of CD47 expression in UM-SCC-17B cells following transfection with CD47-specific siRNA (siCD47) alone or together with GV1001 exposure (100 μg/mL). (**F**) Representative immunofluorescence images of co-cultures of macrophages and UM-SCC-17B cells transfected with control siRNA (siControl) or siCD47. Nuclei were counterstained with DAPI (blue). Cancer cells were labeled with EGFP (green), and macrophages were stained with an anti-F4/80 antibody (red). (**G**) Quantification of macrophage-mediated phagocytosis of UM-SCC-17B cells per field, determined using ImageJ software. All data are presented as the mean ± SEM. Statistical analyses were performed using one-way ANOVA followed by Tukey’s post hoc test (ns, not significant; **** *p* < 0.0001). All experiments were performed with *n* = 5 independent biological replicates.

To determine whether the effect of GV1001 on CD47 expression is conserved in human OSCC, multiple cell lines, including UM-SCC-17B, UM-SCC-1 and FaDu, were examined. As shown in Figure 2A,B, treatment with GV1001 markedly reduced CD47 expression with significant suppression observed at a concentration of 100 μg/mL. The functional consequence of CD47 downregulation was assessed by co-culturing activated macrophages with GV1001-pretreated UM-SCC-17B cells. GV1001-prertreatment significantly enhanced macrophage-mediated phagocytosis of UM-SCC-17B cells (Figure 2C,D). To determine whether this effect was mediated through CD47 inhibition, CD47 expression in UM-SCC-17B cells was reduced by transfection with CD47-specific siRNA (siCD47) (Figure 2E). Comparative analysis revealed similar levels of macrophage-mediated engulfment between GV1001-treated cells and CD47-knockdown cells (Figure 2F,G).

**Figure 3:**
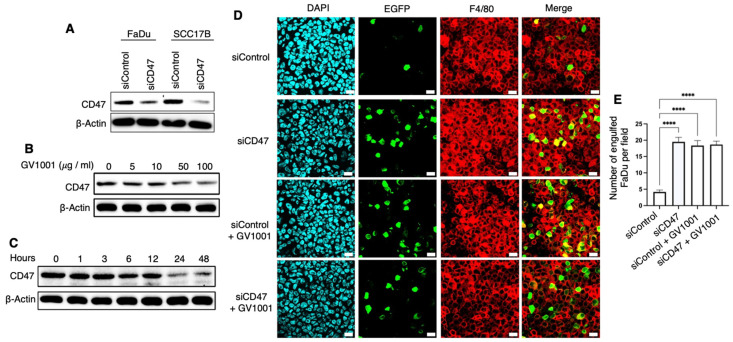
GV1001 hindered CD47 expression and stimulated macrophage-mediated phagocytosis of FaDu cells. (**A**) Western blot analysis of CD47 expression in FaDu cells following transfection with CD47-specific siRNA (siCD47) or siControl. (**B**,**C**) Western blot analysis of CD47 expression in FaDu cells following treatment with GV1001 at the indicated concentrations and time points. β-Actin was used as the loading control. (**D**) Representative immunofluorescence images of co-cultures of macrophages and FaDu cells exposed to GV1001 treatment, siCD47 transfection, or combined GV1001 treatment and siCD47 transfection. (**E**) Quantification of macrophage-mediated phagocytosis of FaDu cells per field, analyzed using ImageJ software. Data are presented as the mean ± SEM. Statistical analyses were performed using one-way ANOVA followed by Tukey’s post hoc test (**** *p* < 0.0001). All experiments were performed using five independent biological replicates.

Consistently, treatment of UM-SCC-17B cells with increasing concentrations of a CD47-blocking antibody resulted in phagocytic activity comparable to that induced by GV1001 treatment (Supplemental Figure S1). Additional human OSCC cell lines were analyzed to confirm the generalizability of these findings. In FaDu cells, CD47 expression was markedly reduced following siCD47 transfection, with UM-SCC-17B cells serving as a control (Figure 3A). Dose- and time-dependent reductions in CD47 expression were observed in both FaDu and UM-SCC-1 cells following GV1001 treatment (Figure 3B,C and Figure S2A,B). Both GV1001 treatment and CD47 silencing significantly enhanced macrophage-mediated engulfment of FaDu cells, with comparable phagocytic activity observed between these two conditions (Figure 3D,E). Similarly, GV1001 treatment significantly increased the phagocytosis of UM-SCC-1 cells by macrophages (Supplemental Figure S2C,D). Collectively, these results demonstrate that GV1001 enhances macrophage-mediated phagocytosis across multiple OSCC models, predominantly through the suppression of CD47 expression, thereby overcoming a key mechanism of tumor immune evasion.

### 2.3. Effects of GV1001 on Expression of CD47 and Pro-Inflammatory Cytokines in MOC1 and UM-SCC-17B Cells

**Figure 4:**
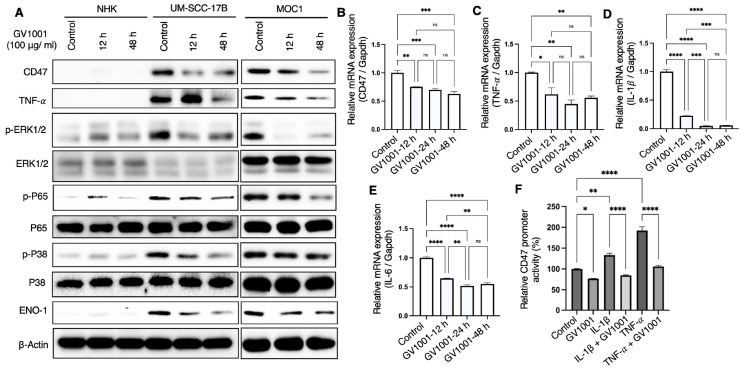
GV1001 suppressed expression of CD47 and its associated signaling molecules in NHK, UM-SCC-17B, and MOC1 cells. (**A**) Western blot analysis of CD47, TNF-α, p-ERK1/2, p-p65, p-p38, and ENO-1 protein levels in NHOK, UM-SCC-17B, and MOC1 cells following treatment with GV1001 (100 μg/mL) for 12 or 48 h. β-Actin was used as the loading control. (**B**–**E**) RT-qPCR analysis of CD47, TNF-α, IL-1β, and IL-6 mRNA expression levels in UM-SCC-17B cells following GV1001 treatment. (**F**) CD47 promoter activity in UM-SCC-17B cells following treatment with IL-1β or TNF-α, alone or in combination with GV1001, as determined by luciferase reporter assay. All data are presented as the mean ± SEM. Statistical analyses were performed using one-way ANOVA followed by Tukey’s post hoc test (ns, not significant; * *p* < 0.05, ** *p* < 0.01, *** *p* < 0.001, **** *p* < 0.0001). All experiments were performed using five independent biological replicates.

To investigate the mechanisms underlying GV100-mediated suppression of CD47, we assessed its effects on signaling molecules known to regulate CD47 expression, including tumor necrosis factor-alpha (TNF-α), phosphorylated extracellular signal-regulated kinase 1/2 (p-ERK1/2), phosphorylated Nuclear factor-kappa B p65 (NF-κB p65) (p-p65), phosphorylated p38 mitogen-activated protein kinase (p-p38 MAPK), and enolase-1 (ENO-1) [24,25,26]. As shown in Figure 4A, GV1001 markedly reduced the levels of TNF-α, p-ERK1/2, p-p38, p-p65, and ENO-1 as well as CD47 in UM-SCC-17B and MOC1 cells in a time-dependent manner, while exerting minimal effects on these protein levels in Normal human keratinocyte (NHK) cells. Consistent with these results, Normal human oral fibroblast (NHOF) cells also showed no appreciable changes in the expression of these proteins following GV1001 treatment (Supplemental Figure S3). Notably, p-p38 was highly expressed in UM-SCC-17B and MOC1 cells, and declined substantially after 48 h of GV1001 treatment, suggesting that this decrease may result from reduced CD47 expression. ENO-1 was also strongly expressed in UM-SCC-17B and MOC1 cells, but nearly absent in NHK cells; GV1001 significantly suppressed ENO-1 in UM-SCC-17B and MOC1 cells. As shown in Figure 4B–E, GV1001 also significantly suppressed gene expression of CD47 and pro-inflammatory cytokines, including TNF-α, Interleukin-6 (IL-6) and Interleukin-1β (IL-1β) in UM-SCC-17B cells. Consistent findings were obtained in MOC1 cells, where GV1001 reduced CD47, TNF-α, and IL-1β gene expression in time- and dose-dependent manners (Supplemental Figure S4A–F).

To further determine whether GV1001 regulated CD47 transcription through altering pro-inflammatory cytokines, UM-SCC-17B cells were transfected with a CD47-luciferase reporter for 24 h and then treated with GV1001 (100 μg/mL), IL-1β (20 ng/mL), IL-1β plus GV1001, TNF-α (100 ng/mL), or TNF-α plus GV1001 for another 48 h. GV1001 markedly attenuated IL-1β- and TNF-α-induced CD47 promoter activity (Figure 4F). Given that GV1001 also suppressed TNF-α and IL-1β expression in cancer cells, its inhibition of CD47 promoter activity might be partially attributable to diminish these pro-inflammatory cytokine levels. Together, these findings indicate that GV1001 downregulates CD47 expression through multiple pathways, including MAPKs- and ENO-1-related signaling and NF-κB-mediated cytokine secretion. This integrated regulatory effect highlights the central role of inflammatory signaling in maintaining CD47 expression and identifies GV1001 as a modulator of this axis in tumor cells.

### 2.4. Effect of GV1001 on Tumor Growth of MOC1 Syngeneic Graft In Vivo

**Figure 5:**
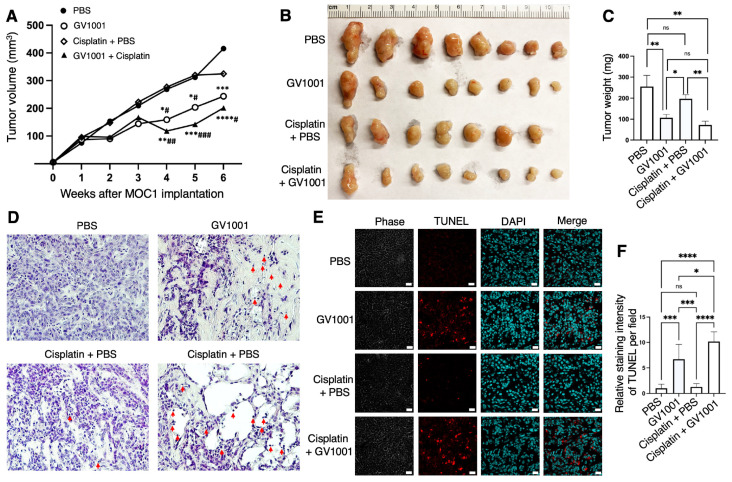
GV1001 alone or in combination with cisplatin suppressed tumor growth in MOC1 syngeneic mouse model in vivo. (**A**) Tumor volume of MOC1 syngeneic graft in mice treated with PBS, GV1001, cisplatin alone, or in combination with GV1001 for 5 weeks. Data were presented as mean ± SEM (*n* = 8 per group). Compared with the control group: * *p* < 0.05, ** *p* < 0.01, *** *p* < 0.001, **** *p* < 0.0001; compared with the Cisplatin group: # *p* < 0.05, ## *p* < 0.01, ### *p* < 0.001. (**B**) Images of harvested tumors from each group at the experimental endpoint. (**C**) Quantification of average tumor weights from each treatment groups. Compared with the control group: * *p* < 0.05, ** *p* < 0.01, ns, not significant. (**D**) Representative hematoxylin and eosin (H&E)-stained tumor sections illustrating histopathological features. Red arrows indicate nuclear condensation. Magnification is 200×. (**E**) Representative TUNEL staining images showing apoptotic nuclei (red) and DAPI-stained nuclei (blue) in tumor tissues from each group. Scale bar = 20 µm. (**F**) Quantification of TUNEL fluorescence intensity analyzed with ImageJ software. All data are presented the mean ± SEM (*n* = 7 or 8). Statistical significance was determined by one-way ANOVA followed by Tukey’s post hoc test (ns **=** not significant, * *p* < 0.05, *** *p* < 0.001, and **** *p* < 0.0001).

To evaluate the in vivo effects of GV1001 on tumor growth, MOC1 cells were engrafted into 8-week-old male C57BL/6 mice to establish syngeneic MOC1 tumors. One week post-implantation, mice were treated with phosphate-buffered saline (PBS), GV1001, cisplatin alone, or a combination of GV1001 and cisplatin. Analysis of tumor growth revealed that cisplatin alone did not significantly reduce tumor volume compared with the control group, whereas GV1001 alone or GV1001 plus cisplatin treatments obviously suppressed tumor growth (Figure 5A). Consistent with these findings, tumor weight measured at the experimental endpoint showed only a modest, non-significant reduction in the cisplatin-treated group compared with PBS controls. In contrast, GV1001 significantly reduced tumor weight, and combined treatment with GV1001 and cisplatin resulted in a more pronounced decrease in tumor weight relative to both the control and cisplatin-alone groups (Figure 5B,C). Histopathological analysis further supported these antitumor effects. Tumors treated with GV1001, either alone or in combination with cisplatin, exhibited a marked increase in necrotic and degenerating tumor cells, characterized by shrunken, fragmented, or swollen morphology and pyknotic or karyorrhectic nuclei. Extensive, disorganized necrotic regions and a substantial reduction in viable tumor cell density were observed in the GV1001-treated groups (Figure 5D). In addition, terminal deoxynucleotidyl transferase–mediated dUTP nick-end labeling (TUNEL) staining revealed increased tumor cell apoptosis in tumors treated with GV1001 alone or in combination with cisplatin (Figure 5E,F). Collectively, these results demonstrate that GV1001 significantly suppresses MOC1 syngeneic graft tumor growth in vivo and enhances tumor cell necrosis and apoptosis. Notably, GV1001, particularly when combined with cisplatin, exhibited superior antitumor efficacy compared with cisplatin alone. As shown in Supplemental Figure S5A, cisplatin treatment resulted in a marked reduction in body weight, whereas GV1001 alone did not significantly affect body weight. Although GV1001 did not initially prevent cisplatin-induced weight loss, body weight gradually recovered during the 6-week period after MOC1 implantation in mice receiving GV1001.

### 2.5. Effects of GV1001 Alone or in Combination with Cisplatin on CD47 Expression and Tumor Cell Necrosis in MOC1 Syngeneic Tumor Model

**Figure 6:**
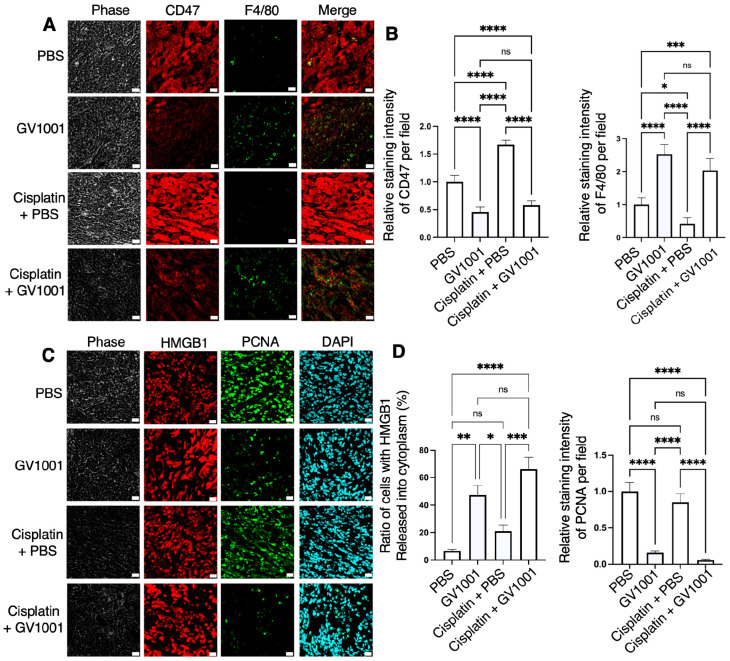
GV1001 treatment suppressed CD47 expression and promoted tumor cell necrosis in MOC1 syngeneic graft tumor. (**A**) Representative immunofluorescence images showing CD47 (red) and F4/80 (green) expression in tumor sections from mice treated with PBS, GV1001, cisplatin + PBS, or cisplatin + GV1001. Scale bars = 20 μm. (**B**) Quantification of relative staining intensities of CD47 and F4/80 per field, analyzed using ImageJ software. (**C**) Representative immunofluorescence images showing HMGB1 (red), PCNA (green), and DAPI (blue) in tumor tissues. Scale bars = 20 μm. (**D**) Quantification of the percentage of tumor cells exhibiting HMGB1 translocation from the nucleus to the cytoplasm and extracellular space, as well as relative PCNA staining intensity per field, analyzed using ImageJ software. All data are presented as the mean ± SEM (*n* = 7 or 8 per group). Statistical significance was determined using one-way ANOVA followed by Tukey’s post hoc test (ns, not significant; * *p* < 0.05, ** *p* < 0.01, *** *p* < 0.001, and **** *p* < 0.0001).

As shown in Figure 6A,B, CD47 was highly expressed in MOC1-derived tumor tissues. Treatment with GV1001 significantly reduced CD47 protein expression and diminished its mRNA levels (Supplemental Figure S6A). Moreover, GV1001 attenuated cisplatin-induced upregulation of CD47 expression and promoted macrophage accumulation within the tumor microenvironment, as evidenced by increased F4/80 immunoreactivity in tumor sections (Figure 6A,B). Tumor cell proliferation, assessed by Proliferating cell nuclear antigen (PCNA) staining, was markedly reduced following GV1001 treatment, with a comparable reduction observed in tumors treated with GV1001 alone or in combination with cisplatin (Figure 6C,D).

Necrotic tumor cells are known to release High mobility group box 1 (HMGB1) protein from the nucleus into the cytoplasm and extracellular space, serving as a Damage-associated molecular pattern (DAMP) signal [27,28]. In tumors treated with GV1001 alone or in combination with cisplatin, HMGB1 was redistributed from the nucleus to the cytoplasm and extracellular compartments. In contrast, in PBS- or cisplatin-treated tumors, HMGB1 remained predominantly localized within the nucleus, indicating largely intact tumor cells (Figure 6C,D). We further assessed pro-inflammatory cytokine levels in both MOC1 syngeneic tumor tissues and mouse serum. As shown in Supplemental Figure S6A, Reverse transcription-quantitative polymerase chain reaction (RT-qPCR) analysis revealed that cisplatin treatment markedly increased the expression of TNF-α and IL-1β in MOC1 syngeneic tumors, whereas combined treatment with GV1001 effectively suppressed this cisplatin-induced upregulation. These findings indicate that GV1001 suppresses CD47 expression, enhances macrophage infiltration, promotes tumor cell necrosis, and mitigates cisplatin-induced inflammatory responses in MOC1 syngeneic tumors. Furthermore, Enzyme-linked immunosorbent assay (ELISA) analysis demonstrated that cisplatin significantly elevated serum levels of TNF-α and IL-1β, while GV1001 treatment attenuated these increases (Supplemental Figure S6B).

### 2.6. Effects of GV1001 and Cisplatin on Tumor Growth in UM-SCC-17B Xenograft Model

**Figure 7:**
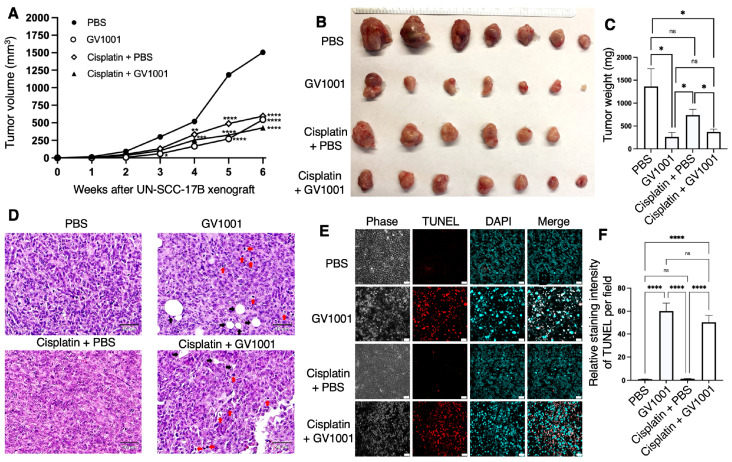
GV1001 treatment inhibited tumor growth of UM-SCC-17B xenografts in vivo. (**A**) Tumor volumes of UM-SCC-17B xenografts in mice treated with PBS, GV1001, cisplatin, or cisplatin plus GV1001 for 5 weeks. (**B**) Images of excised tumors from each treatment group at the experimental endpoint. (**C**) Quantification of tumor weights from each treatment group. (**D**) Representative H&E-stained tumor sections showing histopathological features. Red arrows indicated condensed nuclei of tumor cells, and black arrows implied tissue vacuolization. Magnification is 200×. (**E**) Representative TUNEL staining images showing apoptotic nuclei (red) and DAPI-stained nuclei (blue) in tumor sections. Scale bar = 20 μm. (**F**) Quantification of relative TUNEL fluorescence intensity per field, analyzed using ImageJ software. All data are presented as the mean ± SEM (*n* = 6 or 7 per group). Statistical significance was determined using one-way ANOVA followed by Tukey’s post hoc test (ns, not significant, * *p* < 0.05, ** *p* < 0.01, *** *p* < 0.001, and **** *p* < 0.0001).

To validate the antitumor efficacy of GV1001 in a human oral tumor context, a UM-SCC-17B xenograft model was employed. As depicted in Figure 7A, tumor growth curves demonstrated that GV1001 treatment, either alone or in combination with cisplatin, significantly inhibited tumor progression in the UM-SCC-17B xenograft model. As shown in Figure 7B,C, at the experimental endpoint, tumors were harvested and weighed to further assess therapeutic efficacy. Quantitative analysis revealed that GV1001 treatment markedly decreased tumor weight, and its combination with cisplatin produced a comparable inhibitory effect. In contrast, cisplatin monotherapy induced only a modest, non-significant reduction in tumor weight relative to PBS group. Histopathological examination further supported these findings. Hematoxylin and eosin (H&E) staining showed that cisplatin alone caused mild histological alterations in UM-SCC-17B tumors compared to those of control group, whereas GV1001 treatment, either alone or in combination with cisplatin, induced pronounced cytoplasmic and tissue vacuolization and condensed nuclei, possibly due to tissue necrosis (Figure 7D).

Consistently, TUNEL staining demonstrated a significant increase in apoptotic tumor cells in tumors treated with GV1001 alone or in combination with cisplatin (Figure 7E,F). Collectively, these results indicate that GV1001 exerts potent antitumor activity in the UM-SCC-17B xenograft model. Notably, GV1001, particularly when combined with cisplatin, enhanced tumor suppression and promoted extensive tumor cell apoptosis and necrosis compared with cisplatin monotherapy in vivo. Moreover, as shown in Supplemental Figure S5B, cisplatin treatment resulted in pronounced reduction in body weight, whereas GV1001 alone had no significant effect on mouse body weight. Although GV1001 did not immediately prevent cisplatin-induced weight loss, body weight gradually recovered following 5-week of GV1001 treatment.

### 2.7. Effect of GV1001 on CD47 Expression and Tumor Cell Necrosis in the UM-SCC-17B Xenograft Model

**Figure 8:**
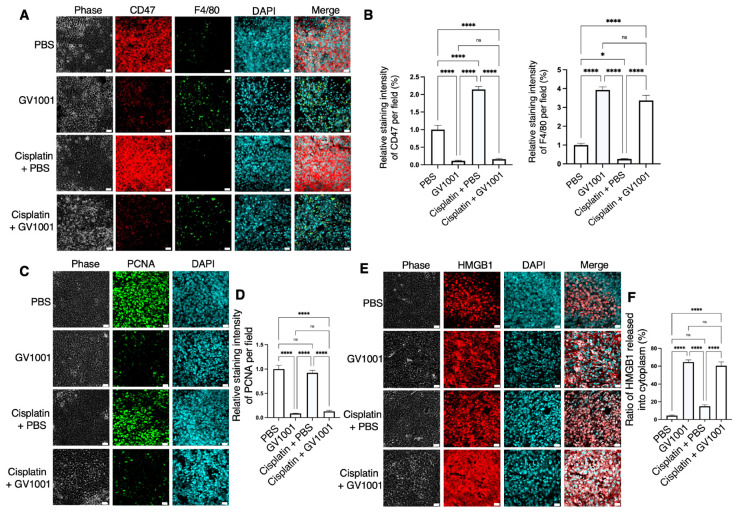
GV1001 mitigated CD47 expression and induced tumor cell necrosis in UM-SCC-17B xenograft tumors. (**A**) Representative immunofluorescence images showing CD47 (red), F4/80 (green), and nuclear staining with DAPI (blue) in UM-SCC-17B xenograft tumors from mice treated with PBS, GV1001, cisplatin plus PBS, or cisplatin plus GV1001. (**B**) Quantification of relative staining intensities of CD47 (left) and F4/80 (right) per field, analyzed using ImageJ software. (**C**) Representative immunofluorescence images showing PCNA (green) and DAPI (blue) staining to assess tumor cell proliferation across treatment groups. (**D**) Quantification of PCNA fluorescence intensity per field, analyzed using ImageJ software. (**E**) Representative immunofluorescence images showing HMGB1 (red) and DAPI (blue) staining to assess tumor cell death. (**F**) Quantification of HMGB1 cytoplasmic localization, expressed as the ratio of HMGB1 fluorescence intensity in the cytoplasm relative to the nucleus, analyzed using ImageJ software. All data are presented as the mean ± SEM (*n* = 6 or 7 per group). Statistical significance was determined using one-way ANOVA followed by Tukey’s post hoc test (ns, not significant, * *p* < 0.05 and **** *p* < 0.0001).

As shown in Figure 8A,B, CD47 expression was highly expressed in UM-SCC-17B xenograft tumors, whereas GV1001 alone substantially downregulated CD47 levels. Cisplatin administration further enhanced CD47 expression; however, GV1001 effectively mitigated this cisplatin-induced upregulation. Consistently, GV1001 treatment alone enhanced macrophage infiltration in tumor tissues, whereas cisplatin distinctly reduced F4/80-positive macrophage accumulation within the tumor microenvironment. Importantly, GV1001 counteracted cisplatin-induced CD47 upregulation and concurrently promoted macrophage accumulation, as evidenced by the increased presence of F4/80-positive cells. These findings suggest that GV1001 promotes a tumor microenvironment more permissive to macrophage-mediated tumor clearance through phagocytosis. ELISA analysis demonstrated that cisplatin significantly increased serum TNF-α and IL-1β concentrations, whereas GV1001 treatment attenuated these cisplatin-induced elevations (Supplemental Figure S6C).

Tumor cell proliferation, assessed by PCNA immunostaining, was significantly reduced following GV1001 treatment, and a comparable inhibitory effect was observed in tumors treated with the GV1001 and cisplatin combination (Figure 8C,D), further supporting the antitumor potential of GV1001. HMGB1 localization was examined to evaluate cell death mechanisms. Treatment with GV1001, either alone or in combination with cisplatin, induced cytoplasmic translocation of HMGB1. In contrast, HMGB1 remained predominantly nuclear in tumors treated with PBS or cisplatin alone, indicating limited cellular damage under those conditions (Figure 8E,F). Collectively, these results indicate that GV1001 exerts multifaceted antitumor effects in a human OSCC xenograft model by downregulating CD47 expression, enhancing macrophage infiltration, suppressing tumor cell proliferation, and promoting tumor cell death in vivo.

## 3. Discussion

CD47 is a multifunctional regulator of tumor biology. Previous studies have demonstrated that CD47 can activate pro-survival pathways, including PI3K/AKT and MAPK/ERK signaling, thereby promoting tumor cell proliferation, migration, and invasiveness [25,29]. In addition, CD47 signaling has been implicated in resistance to apoptosis through modulation of survival-associated proteins and suppression of programmed cell death pathways [30,31]. Beyond its roles in tumor-intrinsic signaling pathways, CD47-mediated immune evasion is increasingly recognized as a major obstacle to effective antitumor immunity, particularly through its role as a “don’t-eat-me” signal that inhibits macrophage-mediated phagocytosis via interaction with signal regulatory protein α (SIRPα) on myeloid cells [32,33]. In oral squamous cell carcinoma (OSCC), elevated CD47 expression has been associated with tumor progression, immune escape, and poor prognosis [34]. In the present study, we demonstrate that GV1001 suppresses CD47 expression in both murine and human OSCC models and enhances macrophage-mediated phagocytosis, identifying a previously unrecognized immunomodulatory mechanism underlying the antitumor activity of GV1001.

A central finding of this study was that GV1001 consistently downregulated CD47 expression across multiple OSCC cell lines, including mouse OSCC (MOC1) and human OSCC cells (UM-SCC-17B, UM-SCC-1, and FaDu), while exerting minimal effects on normal oral keratinocytes. Functionally, this reduction in CD47 expression was translated into a marked increase in macrophage-mediated phagocytosis. Importantly, the magnitude of phagocytosis induced by GV1001 was comparable to that observed following CD47 genetic silencing or antibody-mediated CD47 blockade, strongly supporting the interpretation that CD47 downregulation was a principal driver of GV1001-enhanced macrophage engulfment. The use of multiple human OSCC cell lines were intended to demonstrate that GV1001-induced downregulation of CD47 and enhancement of phagocytosis are broadly applicable across heterogeneous tumor contexts, thereby strengthening the translational relevance of these findings. These findings are consistent with prior studies demonstrating that disruption of CD47–SIRPα signaling restores macrophage phagocytic activity against solid tumors [35,36].

Beyond surface CD47 expression, our data indicate that GV1001 modulates upstream inflammatory and signaling pathways known to regulate CD47 transcription. Pro-inflammatory cytokines such as TNF-α and IL-1β have been shown to induce CD47 expression through NF-κB- and MAPK-dependent mechanisms [24,34,35,37,38]. In this study, GV1001 suppressed TNF-α and IL-1β expression at both the mRNA and protein levels and attenuated NF-κB p65, ERK1/2, and p38 MAPK activation in OSCC cells. Consistent with these observations, GV1001 markedly inhibited IL-1β- and TNF-α-induced CD47 promoter activity, indicating transcriptional regulation. These findings suggest that GV1001 disrupts a feed-forward inflammatory loop in which cytokine signaling sustains CD47 expression and immune evasion. While our findings identify GV1001 as a modulator of CD47 expression through inflammatory signaling, future studies are required to elucidate its upstream molecular targets and the precise regulatory mechanisms governing CD47 expression by GV1001.

The suppression of ENO-1 by GV1001 provides additional mechanistic insight. ENO-1 has been implicated in inflammatory signaling, tumor metabolism, and regulation of immune responses in cancer [39,40]. Its strong expression in OSCC cells and less expression in normal keratinocytes, together with its downregulation by GV1001, suggests that ENO-1 may participate in CD47-associated immune escape pathways. Although the precise relationship between ENO-1 and CD47 requires further investigation, these findings raise the possibility that GV1001 coordinately targets metabolic and inflammatory regulators to suppress CD47 expression.

The in vivo relevance of these mechanisms was demonstrated in both syngeneic and xenograft tumor models. In these systems, GV1001 significantly suppressed tumor growth, enhanced macrophage infiltration, reduced tumor cell proliferation, and promoted tumor cell necrosis and apoptosis. Notably, cisplatin monotherapy led to a non-significantly modest inhibition of tumor growth and instead increased CD47 expression and systemic inflammatory cytokine levels. These observations are consistent with previous reports showing that chemotherapy can paradoxically promote immune evasion and inflammatory signaling within the tumor microenvironment [41,42]. GV1001 effectively counteracted cisplatin-induced CD47 upregulation and inflammatory cytokine release, suggesting that it may restore innate immune surveillance compromised by cytotoxic therapy. A particularly notable finding was the redistribution of HMGB1 from the nucleus to the cytoplasm and extracellular space in GV1001-treated tumors. HMGB1 release is a hallmark of necrotic and immunogenic cell death and serves as a damage-associated molecular pattern that promotes innate immune activation [43,44]. GV1001 leading to the concomitant increase in macrophage infiltration, reduction in CD47 expression, and augmentation of tumor cell death suggest that GV1001 promotes tumor cell elimination, potentially through the enhancement of antitumor immune responses. Furthermore, GV1001 treatment, either as a monotherapy or in combination with cisplatin, resulted in increased TUNEL staining intensity in tumor tissues, indicating elevated DNA fragmentation associated with apoptotic cell death and phagocytic clearance of tumor cells. These findings are consistent with the observations obtained from cultured OSCC cells in vitro.

Despite the promising findings of this study, several limitations should be acknowledged. First, although multiple OSCC cell lines were utilized to enhance the generalizability of GV1001′s function, these in vitro models cannot fully recapitulate the complexity and heterogeneity of human tumors, which may influence responsiveness to GV1001. Second, while both syngeneic and xenograft models were employed, the in vivo systems used in this study remain limited in their ability to comprehensively evaluate immune-mediated mechanisms. In particular, the human xenograft model lacks a fully functional immune system, thereby restricting the assessment of GV1001 effects on adaptive immunity and broader tumor–immune interactions. Although the syngeneic model partially addresses this limitation, additional immune-competent models, including genetically engineered mouse models or orthotopic tumor models, are needed to more accurately define the role of GV1001 in OSCC tumor initiation and progression. Finally, the present study does not fully explore the broader immunological and tumor-intrinsic effects of GV1001. Further studies incorporating diverse in vivo models and comprehensive immune profiling will be necessary to better understand the full therapeutic potential and mechanism of action of GV1001 in OSCC.

In conclusion, this study identifies GV1001 as a novel regulator of CD47 expression and macrophage-mediated phagocytosis in OSCC. By suppressing inflammatory signaling pathways that drive CD47 expression and promoting innate immune-mediated tumor clearance, GV1001 exerts potent antitumor effects in vitro and in vivo. These findings expand the mechanistic understanding of GV1001 and support its potential as an immunomodulatory therapeutic agent targeting innate immune evasion in OSCC.

## 4. Materials and Methods

### 4.1. Cell Cultures

Primary normal human oral keratinocytes (NHK) and normal human oral fibroblasts (NHOF) were established from discarded oral mucosal tissues obtained without patient identifiers or medical information under an Institutional Review Board exemption from University of California, Los Angeles (UCLA), as described previously [45,46]. The mouse OSCC cell line MOC1 was obtained from Kerafast Inc. (cat. no. EWL001-FP, Boston, MA, USA). human OSCC cell lines, including UM-SCC-17B (MilliporeSigma, cat. no. SCC075, St. Louis, MO, USA), UM-SCC-1 (MilliporeSigma, cat. no. SCC070), FaDu (ATCC, cat. no. HTB-43, Manassas, VA, USA), human embryonic kidney 293T cells (HEK293T, ATCC, cat. no. CRL-3216), NHOF and MOC1 were maintained in Dulbecco’s Modified Eagle’s Medium (DMEM; Invitrogen, cat. no. 11995, Carlsbad, CA, USA) supplemented with 10% fetal bovine serum (FBS; Thermo Fisher Scientific, cat. no. 10437028, Waltham, MA, USA). THP-1 human monocytic cells (ATCC, cat. no. TIB-202) were differentiated to macrophages by treatment with 100 nM phorbol 12-myristate 13-acetate (PMA; MilliporeSigma, cat. no. P1585) for 24 h. Following differentiation, cells were washed with PBS and cultured in PMA-free medium for an additional 24 h to allow recovery and maturation. Differentiated macrophages were maintained in RPMI 1640 medium (Thermo Fisher Scientific, cat. no. 11875) supplemented with 10% FBS and were used for subsequent phagocytosis assays as desribed previously [47]. NHK cells were cultured in EpiLife™ Medium (Thermo Fisher Scientific, cat. no. MEPI500CA) supplemented with Human Keratinocyte Growth Supplement (HKGS, Thermo Fisher Scientific, cat. no. S0015). Culture media were replaced every 2 days. Cells were maintained at 37 °C in a humidified atmosphere containing 5% CO_2_.

### 4.2. Treatment of Cells with GV1001, CD47 siRNA, or CD47 Monoclonal Antibody

To evaluate the time- and dose-dependent effects of GV1001 on CD47 expression and macrophage-mediated phagocytosis, oral cancer cells were cultured in the presence of GV1001 (0–100 μg/mL) for 0–48 h prior to harvesting. GV1001 was provided by GemVax/Kael, Inc. (Seongnam-si, Republic of Korea). For CD47 knockdown experiment, cells were were transfected with 80 picomole (pM) of CD47 small interfering RNA (siCD47, Santa Cruz Biotechnology, cat. no. SC-35006; Dallas, TX, USA) or control siRNA (siControl, Santa Cruz Biotechnology, cat. no. SC-37007), according to the manufacturer’s instructions. For antibody blockade assay, cells were cultured in medium containing 2 or 10 μg/mL of CD47 monoclonal antibody purchased from MedChemExpress (cat. no. HY-P99029; Monmouth Junction, NJ, USA) for 2 days, as previously described [48].

### 4.3. Preparation of Lentivirus Vector Expressing Enhanced Green Fluorescent Protein (EGFP)

Lentiviral vectors encoding EGFP were generated to label oral cancer cells. Human embryonic kidney 293T cells at 80–90% confluence in 100 mm culture dishes were co-transfected with 6 μg of pLentiPuro-EGFP-Firefly luciferase plasmid (Addgene, cat. no. 119816, Watertown, MA, USA), 4 μg of lentiviral packaging plasmid psPAX2 (Addgene, cat. no. 12260), and 2 μg of envelope plasmid pMD2.G (Addgene, cat. no. 12259) using 36 μL of Lipofectamine^®^ 2000 (Thermo Fisher Scientific, cat. no. 11668) for 6 h. The transfection medium was then replaced with fresh DMEM supplemented with 10% FBS, and cells were incubated for an additional 48–72 h. Culture supernatants containing lentiviral particles were collected, centrifuged at 1000× *g* for 10 min to remove cell debris, and filtered through 0.45-μm pore-size filters. The filtrates were subsequently concentrated by ultracentrifugation at 10,000× *g* for 30 min. The viral pellet was resuspended in 200 μL PBS and stored at −80 °C [49]. MOC1 and human OSCC cells (UM-SCC-17B, FaDu, and UM-SCC-1) were transduced with 20 μL of the concentrated lentiviral preparation to establish stable EGFP-expressing cell lines. Transduced cells were selected with 4 μg/mL puromycin (MilliporeSigma, cat. no. P8833) for 2 weeks, and surviving colonies were expanded for subsequent macrophage-mediated phagocytosis assays.

### 4.4. In Vitro Macrophage-Mediated Phagocytosis Assay

THP-1-derived differentiated macrophages were incubated in serum-free RPMI-1640 medium for 2 h prior to the addition of 2 × 10^5^ EGFP-labeled cancer cells. Cancer cells were pretreated with GV1001 (100 μg/mL), CD47-specific siRNA (siCD47; 80 pM), or a CD47 monoclonal antibody (2 or 10 μg/mL) for 2 days before coculture with macrophages. Following coculture for 0.5 or 2 h at 37 °C, non-adherent cells were removed by washing three times with PBS, and the remaining cells were fixed with 4% paraformaldehyde for 10 min. Macrophages were identified by immunofluorescent staining with an antibody against the macrophage marker F4/80 (Abcam, cat. no. ab6640, Waltham, MA, USA), followed by incubation with an Alexa Fluor^®^ 594-conjugated secondary antibody (Thermo Fisher Scientific, cat. no. A11007) for 1 h. Nuclei were counterstained with DAPI (Cell Signaling Technology, cat. no. 4083, Danvers, MA, USA). Fluorescent images were acquired using a confocal laser-scanning microscope (LSM 700; Carl Zeiss, Dublin, CA, USA), and the number of EGFP-positive cancer cells engulfed by macrophages was quantified using ImageJ software (v1.54) from the National Institutes of Health [50,51].

### 4.5. Animal Experiments and Animal Welfare

To evaluate the in vivo effects of GV1001 on tumor growth, a syngeneic MOC1 tumor model was established in mice. A total of 32 male C57BL/6J mice (8 weeks of age; The Jackson Laboratory, cat. no. 000664, Bar Harbor, ME, USA) were used for the experiments. Mice were housed under specific pathogen-free conditions with ad libitum access to food and water, as previously described [13,14], MOC1 cells (2 × 10^6^ cells per mouse) were injected subcutaneously into the right flank of each mouse to establish tumors. Seven days after tumor implantation, when tumor volumes reached approximately 50 mm^3^, mice were randomly assigned to four experimental groups (*n* = 8 per group):

1. Control group: Mice received PBS intraperitoneally (i.p.) three times per week for 5 weeks.
2. GV1001 group: Mice were treated with GV1001 (2 mg/kg, i.p.) three times per week for 5 weeks. This dosage has been shown to effectively suppress inflammation without inducing toxicity in mice [12].
3. Cisplatin + PBS group: Mice received cisplatin (2.5 mg/kg, i.p.; Selleck Chemicals, cat. no. S1166, Houston, TX, USA) twice per week for 5 weeks, in combination with PBS administered i.p. three times per week for the same duration. This cisplatin dose has been reported to exert antitumor activity with minimal mortality in mice [52].
4. Cisplatin + GV1001 group: Mice were treated with cisplatin (2.5 mg/kg, i.p.) twice per week for 5 weeks in combination with GV1001 (2 mg/kg, i.p.) administered three times per week for 5 weeks.

To further evaluate the in vivo effects of GV1001 on the tumor growth in a human OSCC xenograft model, a total of 28 eight-week-old male nude mice (Foxn1^nu^, cat. no. 002019; Jackson Laboratory) were used as described above. Homozygous nude mice were selected because they retained functional monocyte and macrophage population while lacking T cells and exhibiting partial B-cell deficiencies, thereby permitting assessment of GV1001-mediated antitumor effects in the context of macrophage-dependent phagocytic activity. UM-SCC-17B cells (2 × 10^6^ per mouse) were injected subcutaneously into the right flank to establish xenografts. Seven days after cell injection, mice were randomized into four groups (*n* = 7 per group) and treated according to the same dosing regimen described above. Animal health, behavior, and general appearance were monitored daily, and body weights were recorded weekly throughout the study. At the end of the 5-week treatment period, mice were humanely euthanized. Prior to euthanasia, animals were anesthetized with ketamine (100 mg/kg) and xylazine (5 mg/kg) to minimize discomfort and stress.

### 4.6. Sample Collection

One week after completion of the 5-week treatment period, mice were anesthetized and whole blood was collected by cardiac puncture, followed by harvest of the tumor tissues. Each tumor specimen was divided into two portions: one portion was immediately snap-frozen in liquid nitrogen and stored at −80 °C for RNA extraction and subsequent analysis of pro-inflammatory cytokine gene expression, while the remaining portion was fixed in 4% paraformaldehyde for H&E staining and immunofluorescence analysis. Collected blood samples were centrifuged at 2000× *g* for 15 min to separate serum, which was subsequently used for the quantification of circulating pro-inflammatory cytokine levels.

### 4.7. H&E and Immunofluorescence Staining (IF) Assays

Paraffin-embedded tumor sections were stained with H&E to assess tissue histopathology. For IF analysis, paraffin-embedded tumor tissue sections and fixed OSCC cells were incubated overnight at 4 °C with primary antibodies against F4/80 (Abcam, cat. no. ab6640), CD47 (Abcam, cat. no. ab284132), PCNA (Santa Cruz Biotechnology, cat. no. sc-56), or HMGB1 (Cell Signaling Technology, cat. no. 3935). Following primary antibody incubation, samples were incubated with Alexa Fluor^®^ 488- or Alexa Fluor^®^ 594-conjugated secondary antibodies (Thermo Fisher Scientific, cat. nos. A11029 and A11010, respectively) for 1 h at room temperature. Slides and cells were mounted using VECTASHIELD^®^ Antifade Mounting Medium with DAPI (Vector Laboratories, cat. no. H1200, Newark, CA, USA). Fluorescent images were acquired using a confocal laser-scanning microscope (LSM 700; Carl Zeiss). All staining procedures were performed according to the manufacturers’ instructions and previously published protocols [13]. Image analysis was conducted using ImageJ software. Background fluorescence was subtracted using a rolling-ball radius of 50 pixels, and thresholding was applied uniformly across all images within each experiment [53]. Mean fluorescence intensity was normalized to the corresponding DAPI signal within the same field. All image quantifications were performed in a blinded manner with respect to experimental conditions.

### 4.8. Apoptosis Assay

Apoptotic cells in mouse tumor tissues were identified by terminal deoxynucleotidyl transferase–mediated dUTP nick-end labeling (TUNEL) using a commercially available assay kit (Cell Signaling Technology, cat. no. 25879), according to the manufacturer’s instructions.

### 4.9. Reverse Transcription-Quantitative Polymerase Chain Reaction (RT-qPCR)

Total RNA was isolated from cultured cells and mouse tumor tissues using the RNeasy Micro Kit (Qiagen, cat. no. 74004, Valencia, CA, USA). Reverse transcription was performed using the SuperScript™ III First-Strand Synthesis Kit (Thermo Fisher Scientific, cat. no. 18080044) under the following conditions: 65 °C for 5 min, 25 °C for 2 min, and 45 °C for 60 min. Quantitative PCR was carried out using PowerUp™ SYBR™ Green Master Mix (Thermo Fisher Scientific, cat. no. A25742) according to the manufacturer’s instructions. Primer sequences were obtained from the Universal Probe Library (Roche Diagnostics, South San Francisco, CA, USA). The PCR cycling conditions consisted of an initial denaturation at 95 °C for 2 min, followed by 40 cycles of denaturation at 95 °C for 15 s and annealing/extension at 60 °C for 1 min. The sequences of the primers used for RT-qPCR are listed in Table S1. Glyceraldehyde 3-phosphate dehydrogenase (GAPDH) was used as internal control using human or mouse GAPDH primers, and the fold induction was calculated using the comparative ΔCq method and were presented as relative transcript levels (2^−ΔΔcq^) [54].

### 4.10. Western Blot Analysis

Whole-cell lysates were prepared from cultured cells using radioimmunoprecipitation assay (RIPA) buffer (Thermo Fisher Scientific, cat. no. 89901). Proteins were resolved by sodium dodecyl sulfate–polyacrylamide gel electrophoresis (SDS-PAGE) and transferred onto Immobilon^®^-P polyvinylidene difluoride (PVDF) membranes (MilliporeSigma, cat. no. IPVH00010). Membranes were incubated overnight at 4 °C, with primary antibodies, followed by horseradish peroxidase-conjugated secondary antibodies for 1 h at room temperature. Protein bands were detected using Clarity™ Western Enhanced chemiluminescence (ECL) substrate, (Bio-Rad, cat. no. 1705061, Hercules, CA, USA), as previously described [11]. The following primary antibodies were used: CD47 (Abcam, cat. no. ab284132), TNF-α (Abcam, cat. no. ab6671), NF-κB p65 (Santa Cruz Biotechnology, cat. no. sc-8008), p-p38 (Cell Signaling Technology, cat. no. 9211), total p38 (Cell Signaling Technology, cat. no. 9212), p-p65 (Cell Signaling Technology, cat. no. 3036), p-ERK1/2 (Cell Signaling Technology, cat. no. 4370), total ERK1/2 (Cell Signaling Technology, cat. no. 4695) and ENO1 (Cell Signaling Technology, cat. no. 3810), as well as β-actin (Cell Signaling Technology, cat. no. 4970), which is served as a loading control.

### 4.11. Enzyme-Linked Immunosorbent Assay (ELISA)

Serum levels of interleukin-6 (IL-6), IL-1β, and TNF-α in mice were quantified using a commercially available ELISA kit (Invitrogen, Carlsbad, CA, USA), according to the manufacturer’s instructions, as previously described [55].

### 4.12. CD47 Promoter Activity Assay

The −897 to +17 bp region of the human CD47 promoter was amplified and cloned into the pGL3-Basic luciferase reporter vector (Addgene, cat. no. 212936) to generate a firefly luciferase reporter construct, as previously described [56]. The following primers were used for cloning: forward primer, 5′-GGGGGTACCATGTTTACCACCGTGAAT GG-3′; and reverse primer, 5′-GGGAAGCTTCTACCAGGGGCCACATCTCC-3′. UM-SCC-17B cells were transfected with 1 µg of the CD47 promoter–luciferase construct using 3 µL of Lipofectamine™ 2000 reagent (Thermo Fisher Scientific, cat. no. 11668), together with 50 ng of the pRL-SV40 vector (Promega, cat. no. E2231, Madison, WI, USA) encoding renilla luciferase as an internal control. Cell lysates were harvested, and dual-luciferase activity was measured using the Dual-Luciferase Reporter Assay System (Promega, cat. no. E1910, Madison, WI, USA) according to the manufacturer’s instructions. Relative luciferase activity was calculated as the ratio of firefly to renilla luciferase activity and expressed as CD47 promoter activity, as previously described [57].

### 4.13. Statistical Analyses

All statistical analyses were performed using GraphPad Prism 9 (GraphPad Software, Boston, MA, USA). Comparisons among multiple groups were conducted using one-way ANOVA followed by Tukey’s post hoc test. A *p*-value < 0.05 was considered statistically significant. All in vitro experiments were independently performed at least five times, and data are presented as the mean ± SEM.

## 5. Conclusions

This study demonstrates that GV1001 suppresses CD47 expression in both murine and human oral squamous cell carcinoma cells, thereby enhancing macrophage-mediated phagocytosis of tumor cells. Mechanistically, GV1001 inhibits CD47 transcription through the suppression of pro-inflammatory cytokines and MAPK/NF-κB-related signaling pathways. In vivo, GV1001 significantly inhibited tumor growth, promoted tumor cell necrosis and apoptosis, and enhanced macrophage infiltration in both syngeneic and xenograft tumor models. Moreover, GV1001 counteracted cisplatin-induced CD47 upregulation and inflammatory responses, highlighting its potential as a promising immunomodulatory therapeutic strategy for OSCC, particularly in combination with conventional chemotherapy.

## Data Availability

The data presented in this study are available on request from the corresponding author due to privacy.

## Supplementary Materials

The following supporting information can be downloaded at: https://www.mdpi.com/article/10.3390/ijms27073340/s1.

## Abbreviations

The following abbreviations are used in this manuscript:

CD47	Cluster of Differentiation 47
hTERT	Human telomerase reverse transcriptase
SIRPα	Signal regulatory protein α
HSP70/90	Heat shock protein 70/90 Linear dichroism
HIF-1α	Hypoxia-inducible factor-1alpha
MOC1	Mouse oral cancer 1
VEGF	Vascular endothelial growth factor
DAPI	4′,6-diamidino-2-phenylindole
EGFP	Enhanced green fluorescent protein
siCD47	CD47-specific siRNA
TNF-α	Tumor necrosis factor-alpha
p-ERK1/2	phosphorylated extracellular signal-regulated kinase 1/2
p-p38	phosphorylated p38 mitogen-activated protein kinase
p-p65	phosphorylated NF-κB p65
ENO-1	Enolase-1
IL-6	Interleukin-6
IL-1β	Interleukin-1beta
SEM	Standard error of the mean
ANOVA	Analysis of variance
TUNEL	Terminal deoxynucleotidyl transferase–mediated dUTP nick-end labeling
H&E	Hematoxylin and eosin
PCNA	Proliferating Cell Nuclear Antigen
DAMP	Damage-associated molecular pattern
HMGB1	High mobility group box 1
RT-qPCR	Reverse transcription-quantitative polymerase chain reaction
NHK	Normal human oral keratinocyte
NHOF	Normal human oral fibroblast
HEK293T	Human embryonic kidney 293T cells
FBS	Fetal bovine serum
DMEM	Dulbecco’s Modified Eagle’s Medium
PMA	Phorbol 12-myristate 13-acetate
PBS	Phosphate-buffered saline
HKGS	Human Keratinocyte Growth Supplement
i.p.	Intraperitoneally
IF	Immunofluorescence staining
GAPDH	Glyceraldehyde 3-phosphate dehydrogenase
RIPA	Radioimmunoprecipitation assay
SDS-PAGE	Sodium dodecyl sulfate–polyacrylamide gel electrophoresis
PVDF	Polyvinylidene difluoride
ELISA	Enzyme-linked immunosorbent assay
ECL	Enhanced chemiluminescence
