# Supplementary material for: GV1001 Reprograms CD47 Immune Checkpoint to Restore Macrophage Antitumor Activity in Oral Squamous Cell Carcinoma
Source: Int J Mol Sci. 2026 Apr 7;27(7):3340. doi: 10.3390/ijms27073340 (PMC13073131; doi:10.3390/ijms27073340)
Supplement: Supplementary file 1 [file ijms-27-03340-s001.zip › ijms-4222581-supplementary.pdf]

Supplementary Files

GV1001 reprograms CD47 immune checkpoint to restore macrophage antitumor activity in oral squamous cell carcinoma

Wei Chen[1], Seojin Kim[1], Cheyenne Beheshtian[1], Angela Jun[1], Sangjae Kim[4] and No-Hee Park[1,2,3,*]

1 The Shapiro Family Laboratory of Viral Oncology and Aging Research, UCLA School of Dentistry, 714 Tiverton Ave, Los Angeles, CA, 90095, USA

2 UCLA Jonsson Comprehensive Cancer Center, 10833 Le Conte Ave, Los Angeles, 90095, CA, USA

3 Department of Medicine, David Geffen School of Medicine at UCLA, 10833 Le Conte Ave, Los Angeles, CA, 90095, USA

4 Teloid Inc., 920 Westholme Avenue, Los Angeles, CA 90024, USA

* Correspondence to: Dr No-Hee Park, The Shapiro Family Laboratory of Viral Oncology and Aging Research, UCLA School of dentistry, 417 Tiverton Avenue, Los Angeles, CA 90095, USA E-mail: nhpark@g.ucla.edu

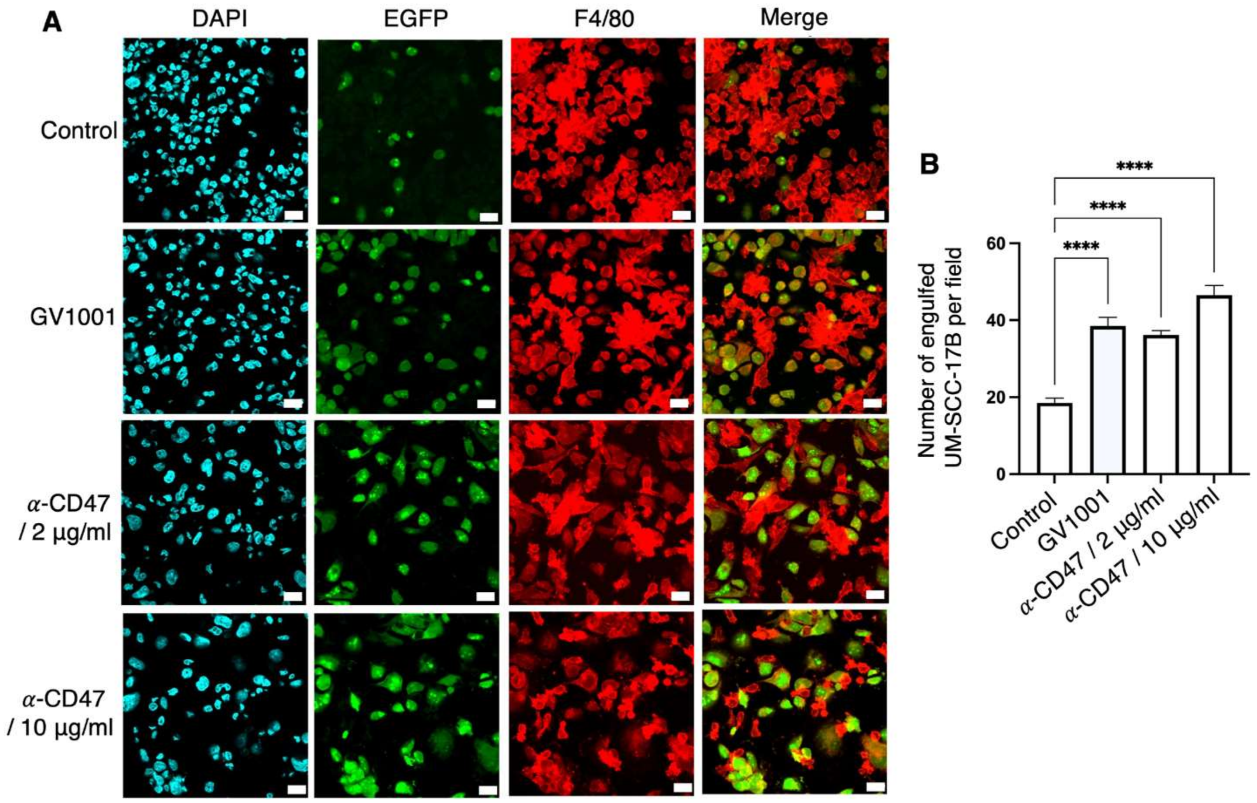

Figure S1. Antibody-mediated CD47 blockade induced macrophage-mediated phagocytosis of UM-SCC-17B. (A) Representative immunofluorescent staining images of co-culture of GV1001- or CD47 antibody-pretreated UM-SCC-17B cells and macrophages following 2 h of incubation. Cell nuclei were stained with DAPI (blue). EGFP fluorescence (green) indicates UM-SCC-17B cancer cells, whereas macrophages were stained red with an anti-F4/80 antibody. **(B)** Quantification of the number of engulfed UM-SCC-17B cells by macrophages per field through ImageJ software. Data are presented the mean ± SEM. Statistical significance was determined by one-way ANOVA followed by Tukey's post hoc test (ns = not significant, and **** $p < 0.0001$).

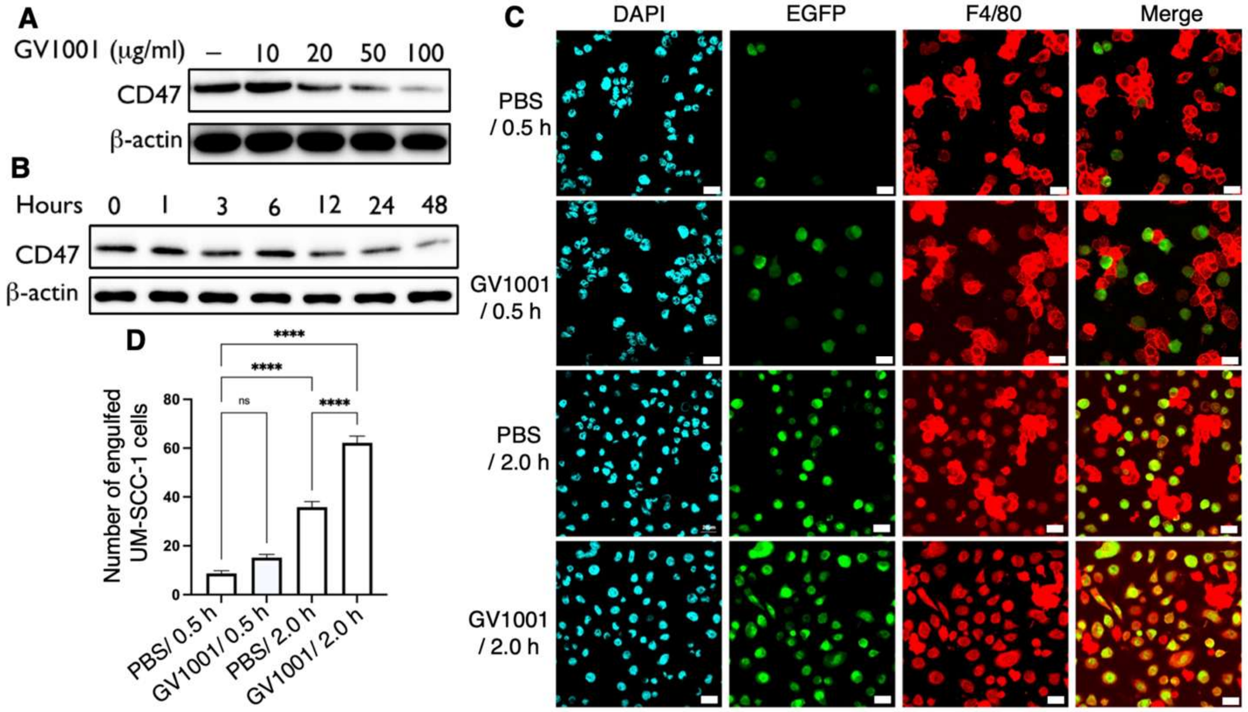

Figure S2. GV1001 inhibited CD47 expression and increased macrophage-mediated phagocytosis of UM-SCC-1 cells. (A, B) Western blot analysis of CD47 expression in UM-SCC-1 cells following treatment with GV1001 at the indicated concentrations and time points. β-Actin was used as a loading control. **(C)** Representative immunofluorescence images of co-cultures of GV1001-pretreated UM-SCC-1 cells and macrophages after 0.5 or 2 h of incubation. Cell nuclei are stained blue with DAPI. Cancer cells were labeled with EGFP (green), and macrophages were stained with an anti-F4/80 antibody (red). **(D)** Quantification of macrophage-mediated phagocytosis of UM-SCC-1 cells per field, determined using ImageJ software. Data are presented as the mean ± SEM. Statistical analysis was performed using one-way ANOVA followed by Tukey's post hoc test (ns, not significant; **** $p < 0.0001$). All experiments were performed in quintuplicate.

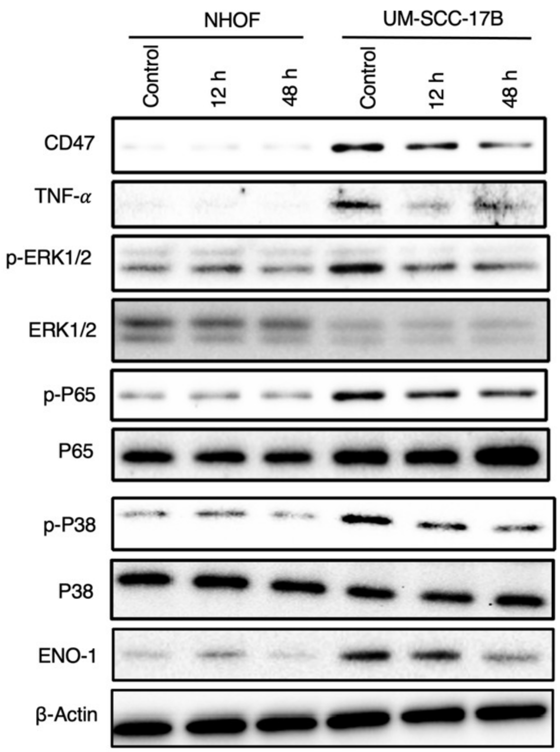

Figure S3. GV1001 inhibited expression of CD47 and its associated signaling molecules in NHOF and UM-SCC-17B cells. Western blot analysis of CD47, TNF-α, phosphorylated ERK1/2 (p-ERK1/2), phosphorylated NF-κB p65 (p-p65), phosphorylated p38 MAPK (p-p38), and enolase-1 (ENO-1) protein levels in NHOF and UM-SCC-17B cells following treatment with GV1001 (100 μg/ml) for 12 or 48 h. β-Actin was used as the loading control.

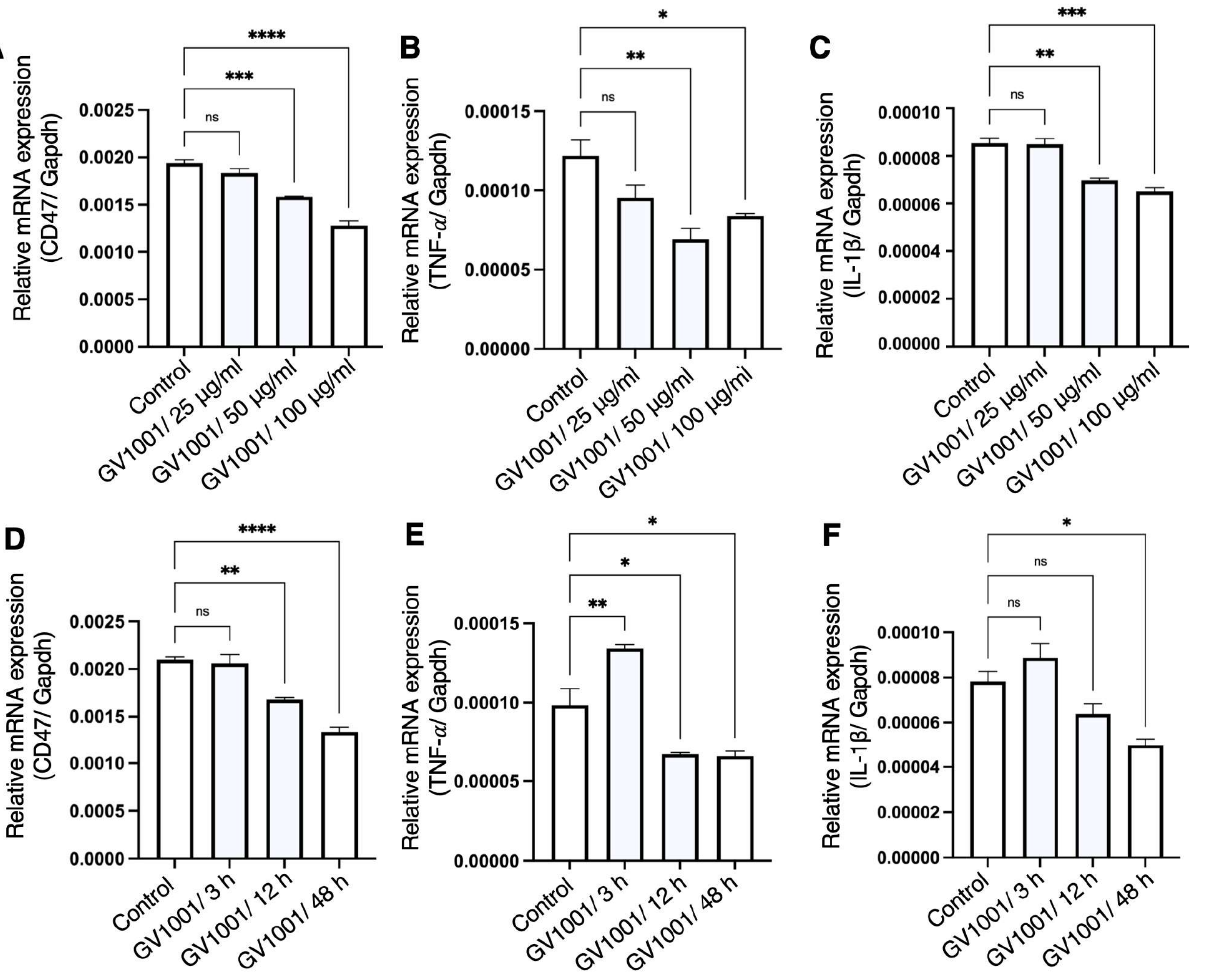

Figure S4. GV1001 repressed gene expression of CD47 and pro-inflammatory cytokines in MOC1 cells. (A, B, C) Effect of GV1001 on the gene expression of CD47, TNF-α and IL-1β in MOC1 in a dose-dependent manner. **(D, E, F)** Effect of GV1001 (100 μg/ml) on the gene expression of CD47, TNF-α and IL-1β in MOC1 in a time-dependent manner. Data are presented the mean ± SEM. Statistical significance was determined by one-way ANOVA followed by Tukey's post hoc test (ns = not significant, * $p < 0.05$, ** $p < 0.01$, *** $p < 0.001$, and **** $p < 0.0001$). All experiments were performed in quintuplicate.

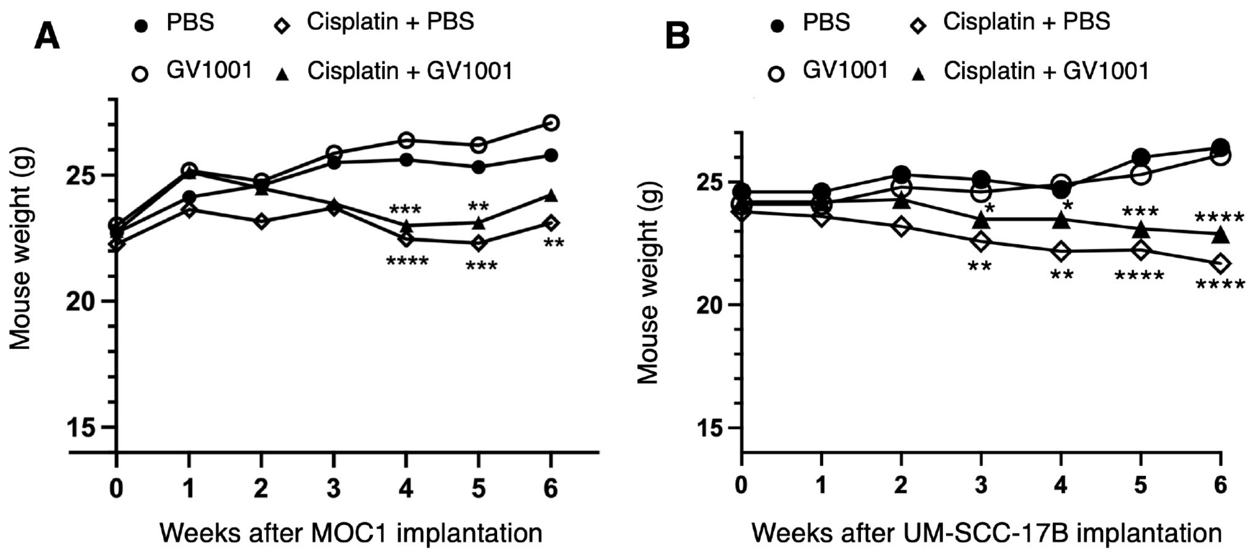

Figure S5. Temporal changes in mouse weight. Body weights of mice with MOC1 syngeneic graft in C57BL/6J mice **(A)** and UM-SCC-17B xenograft in nude mice **(B)** exposed to PBS, GV1001, Cisplatin and Cisplatin + GV1001. Statistical significance was determined by one-way ANOVA followed by Tukey's post hoc test. Comparisons were made with the PBS-treated group: * $p < 0.05$, ** $p < 0.01$, *** $P < 0.001$, and **** $p < 0.0001$.

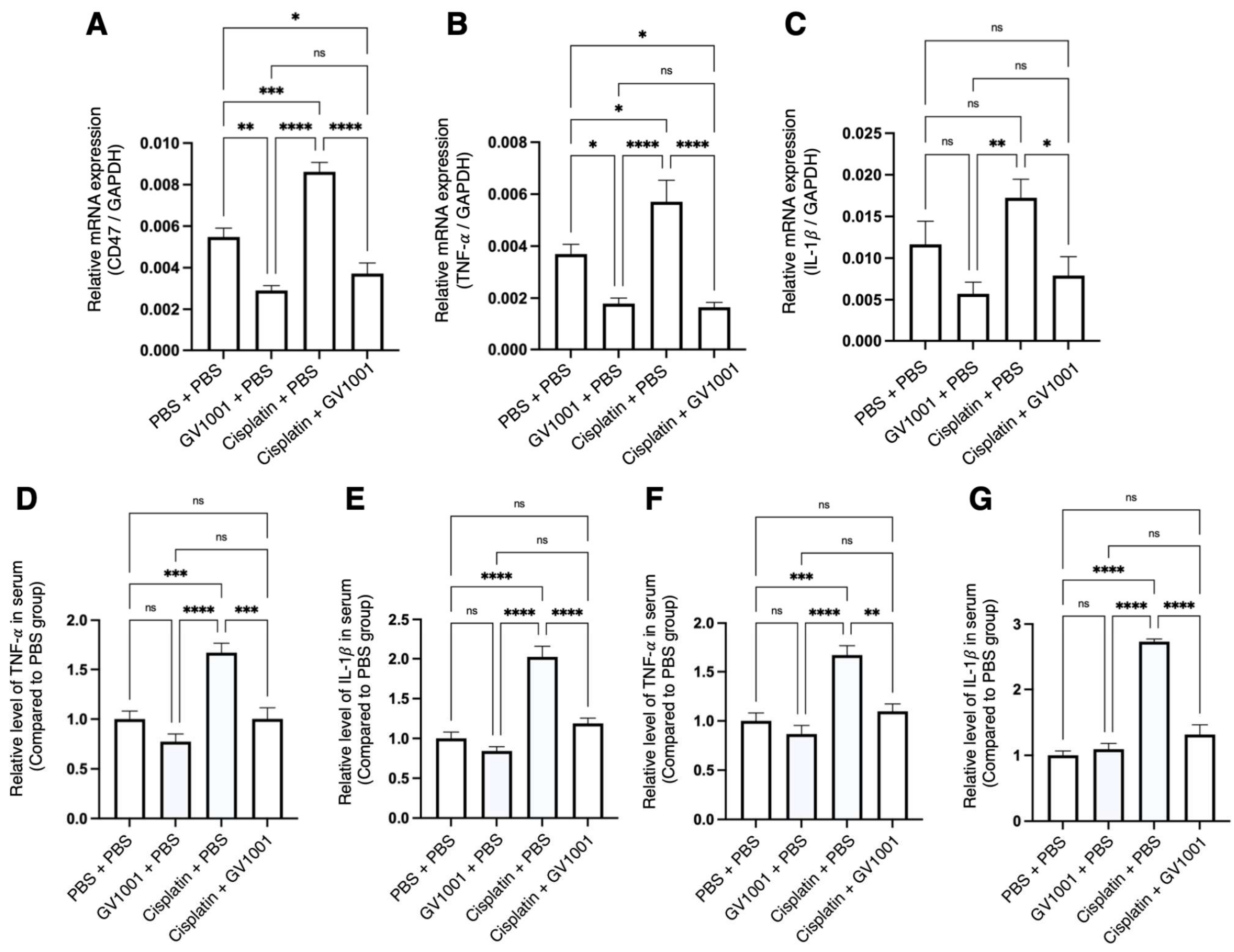

Figure S6. GV1001 mitigated cisplatin-induced pro-inflammatory cytokine expression and secretion in mice with OSCC tumor implantation. (A, B, C) GV1001 suppressed the cisplatin-induced gene expression of CD47, TNF-α and IL-1β in MOC1 syngeneic graft tumor tissues. Gene expression was quantified by RT-qPCR, with GAPDH as the internal control. **(D, E)** ELISA analysis of TNF-α and IL-1β levels in the sera of mice with MOC1 syngeneic graft tumor. **(F, G)** TNF-α and IL-1β levels in the sera of mice implanted with UM-SCC-17B xenograft. Data were presented the mean ± SEM. Statistical significance was determined by one-way ANOVA followed by Tukey's post hoc test (ns = not significant, * $p < 0.05$, and ** $p < 0.01$). All experiments were performed in quintuplicate.

Supplemental Table:

Supplemental Table S1: Sequences of the primers for reverse transcription- quantitative polymerase chain reaction (RT-qPCR).

Genes	Forward primer 5'-3'	Reverse primer 5'-3'
mCD47	GGTGGGAAACTACACTTGCGAAG	CTCCTCGTAAGAACAGGCTGATC
mIL-1β	TGGACCTTCCAGGATGAGGACA	GTTCATCTCGGAGCCTGTAGTG
mTNF-α	TCAGGTTGCCTCTGTCTCAG	GCTCTGTGAGGAAGGCTGTG
mTGF-β1	TGATACGCCTGAGTGGCTGTCT	CACAAGAGCAGTGAGCGCTGAA
mGapdh	AGCTTGTCATCAACGGGAAG	TTTGATGTTAGTGGGGTCTCG
hCD47	TATCCTCGCTGTGGTTGGACTG	TAGTCCAAGTAATTGTGCTAGAGC
hTNF-α	CCAGGGACCTCTCTCTAATCA	TCAGCTTGAGGGTTTGCTAC
hIL-1 β	ATGGACAAGCTGAGGAAGATG	CCCATGTGTCGAAGAAGATAGG
hGapdh	AGCCACATCGCTCAGACAC	GCCCAATACGACCAAATCC
